# Alcohol Involvement in Sexual Behaviour and Adverse Sexual Health Outcomes from 26 to 38 Years of Age

**DOI:** 10.1371/journal.pone.0135660

**Published:** 2015-08-12

**Authors:** Jennie L. Connor, Robyn M. Kydd, Nigel P. Dickson

**Affiliations:** Department of Preventive and Social Medicine, Dunedin School of Medicine, University of Otago, Dunedin, New Zealand; Asociacion Civil Impacta Salud y Educacion, PERU

## Abstract

**Background:**

Research on alcohol and sexual behaviour has focused on young adults or high-risk groups, showing alcohol use contributing to riskier sexual choices. Adults now in their late thirties have been exposed to heavier drinking norms than previously, raising questions about effects on sexual wellbeing. We examined self-reported use and consequences of alcohol in sexual contexts, and its association with usual drinking pattern at age 38, and also associations of heavy drinking occasion (HDO) frequency with number of sexual partners, sexually transmitted infections (STIs), and terminations of pregnancy (TOPs), from 26–32 and 32–38 years of age.

**Methods:**

Members of the Dunedin Study birth cohort answered computer-presented questions about sexual behaviour and outcomes, and interviewer-administered alcohol consumption questions, at age 26, 32 and 38 years.

**Results:**

Response level was >90% at each assessment. At 38, drinking before or during sex in the previous year was common (8.2% of men; 14.6% of women reported “usually/always”), and unwanted consequences were reported by 13.5% of men and 11.9% of women, including regretted sex or failure to use contraception or condoms. Frequent heavy drinkers were more likely to “use alcohol to make it easier to have sex” and regret partner choice, particularly women. Heavy drinking frequency was strongly associated with partner numbers for men and women at 32, but only for women at 38. Significantly higher odds of STIs amongst the heaviest drinking men, and TOPs amongst the heaviest drinking women were seen at 32–38.

**Conclusions:**

Alcohol involvement in sex continues beyond young adulthood where it has been well documented, and is common at 38. Women appear to be more affected than men, and heavy drinking is associated with poorer outcomes for both. Improving sexual health and wellbeing throughout the life course needs to take account of the role of alcohol in sexual behaviour.

## Introduction

The relationships between alcohol consumption and sexual behaviour are complex, with empirical support for causal relationships in both directions, and for shared causes of both behaviours, depending on the context and population [1–3]. Apart from selected high risk populations such as those infected with HIV [4], most published research has focused on adolescents and young adults. Among young people, the high prevalence of hazardous alcohol consumption, risk-taking, and sensation-seeking activities has made it difficult to quantify the causal elements of commonly observed associations between alcohol and sexual outcomes [1 5 6]. However, there is substantial evidence for the cognitive effects of alcohol affecting decision-making and resulting in riskier choices regarding sexual activity [1 6–8], and a recent systematic review concluded that blood alcohol concentration is an independent risk factor for the intention to engage in unprotected sex among young adults (mean age 24.0 years) in North American college and community settings [9].

In general populations, the prevalence of hazardous alcohol consumption is typically lower in older age groups [10], along with lower levels of other risk-taking behaviours, and in concert with the formation of long term sexual partnerships. However, cross-sectional surveys do not separate age and cohort effects on alcohol consumption, and changes in the last two decades suggest that alcohol’s role in sexual behaviour beyond young adulthood may have become more relevant to sexual health outcomes.

Adults currently in their late thirties in most English-speaking countries have been exposed to a youth culture where hazardous drinking behaviour has been common from early teenage years [11] and where, at least in the UK, first sexual intercourse is more likely to have been experienced when intoxicated than previously [12]. Young women’s drinking has increased sharply in a single generation [13], and gender convergence of drinking patterns is commonly seen [11 14], with increased drinking among women of all ages during the last two decades [15].

Breakdown of long term relationships, and initiation of new ones, are not uncommon in the 30–40 year age group and there are a number of roles alcohol use might play in these transitions, particularly if there has been a persistence of, or return to, drinking behaviour experienced as a young adult [16]. Among the potential adverse consequences of heavy drinking for sexual health in this age group are those already identified in younger people, such as sexually transmitted infections, unwanted pregnancies, and regretted sexual experiences [17].

Little has been published in the last twenty years describing alcohol use in the context of sexual behaviour of heterosexual adults over 30 in the general population, or quantifying associations of their alcohol use with sexual behaviour and/or health. Most studies have been conducted in non-representative groups such as military personnel [18], men who have sex with men, or STI clinic attenders [19]. One exception is a 1994 population-based cross-sectional study in the U.S. which analysed several drinking measures in relation to being sexually active, having more than one partner in the past year, and condom use [20]. However, no age- or sex-specific findings were reported. Another is the Natsal-2 study in the UK, which compared nationally representative surveys of 16–44 year olds in 2000/1 and 1990/1 with respect to alcohol use at first heterosexual intercourse, and quantified the association of heavier drinking (defined as an average level above current guidelines) with a range of outcomes: numbers of sexual partners, unprotected sex, prevalence of sexually transmitted infections (STIs), sexual function problems, terminations of pregnancy (TOPs) and emergency contraception use [12]. However, this study published age-adjusted rather than age-specific findings, and did not investigate the association of frequency of heavy drinking episodes with sexual risk behaviours or outcomes, even though the implicit mechanism of effect is intoxication.

In this paper we report on alcohol’s role in sexual behaviour and outcomes among 38 year olds from a New Zealand birth cohort. In particular we describe, for men and women separately, 1. self-reported use of alcohol in relation to sexual activity and perceived consequences of that use, 2. cross-sectional associations of heavy drinking frequency with self-reported alcohol involvement in sex, 3. associations of heavy drinking frequency with partner numbers, STIs and TOPs, and 4. whether associations of heavy drinking with these three outcomes have changed since the 26–32 year old period of the same cohort.

## Methods

Participants were members of the Dunedin Multidisciplinary Health and Development Study, a longitudinal study of a complete birth cohort from Dunedin, New Zealand, born between April 1, 1972 and March 31, 1973. The original sample of 1037 children (91% of 1139 eligible births) was first seen at 3 years of age and most recently at 38 years, when 95% of the 1007 living study members were assessed in 2010–2012. Sociodemographic characteristics of the sample are generally representative of the New Zealand population for this age group, with slightly higher educational attainment and fewer people of Māori ethnicity at age 21 [21]. A description of the development of the cohort has been recenty published [22]. Information on sexual and reproductive health was collected using a computerised questionnaire at ages 21, 26, 32 and 38.

Ethical approval for Phase (age) 38 of the Dunedin Multidisciplinary Health and Development Study was granted by the Lower South Regional Ethics Committee of the Ministry of Health [LRS/10/03/012]. Approval specifically included this study [Investigator Nigel Dickson]. Written informed consent was obtained from all participants.

Study members who self-identified as heterosexual and reported mostly opposite sex sexual experiences, at each assessment, were included in the analyses reported in this paper. We restricted the scope to heterosexual study members because we could not assume that the behaviours and associations described would be similar in heterosexual and sexual minority subgroups, and the small numbers in non-heterosexual groups were insufficient to conduct independent analyses. At age 32 and at age 38, current sexual identity was assessed with the question “Do you consider yourself to be…?” followed by a list of options. In another section of the questionnaire the sex of people with whom the participant had had sexual experiences with in the past year was asked by first defining this as “… any contact you felt was sexual … It could have been kissing, touching, or intercourse” then offering options that included only the opposite sex, and mainly the opposite sex [23].

### Measures

#### Self-reported alcohol use in sexual relationships

The computer-presented sexual health and reproductive health questionnaire at age 38 included a series of closed questions about alcohol consumption and sex that had not been asked in earlier assessments. Each participant was asked about the frequency of their own and their sexual partner’s drinking before or during sex in the previous 12 months, the effects of such drinking, and unintended consequences that they attributed to drinking.

#### Number of sexual partners, self-reported STIs, and TOPs

The questions on sexual experiences were based on those used in the 1990 British National Survey of Sexual Attitudes and Lifestyles (Natsal) [24]. At age 32 and 38 assessments, these included questions on the number of opposite and same sex partners in the past 12 months, and in the 6 years since the previous assessment, and whether they had experienced one or more STIs since the previous assessment [25]. Conditions were identified from a list of the common STIs (chlamydia, non-specific urethritis (NSU), genital warts, herpes, gonorrhoea, trichomoniasis, syphilis), or specified as an “other STI”. In addition, we identified study members who had positive serology for herpes simplex virus type 2 (HSV-2) or chlamydia trachomatis which had been negative at the previous assessment. Detailed information was collected on pregnancies of female study members and of partners of male study members. From this we determined whether the study member (or a partner for men) had experienced one or more TOPs between assessments. TOPs for fetal abnormality were excluded from the analysis.

#### Alcohol consumption pattern

Given that the hypothesised mechanism for any causal contribution of alcohol to sexual behaviour and outcomes is intoxication, we considered frequency of heavy drinking occasions (HDOs) to be the measure of drinking pattern most relevant for this analysis. Pattern rather than average volume of consumption has been demonstrated to be the stronger predictor of a range of social consequences of drinking [26]. At each of the three assessments (26,32,38 years), frequency of HDOs in the preceding 12 months was self-reported in the context of a psychiatric diagnostic interview with a trained interviewer, using a definition of HDO as “five or more drinks in one sitting”. We categorised frequencies as “never or less than monthly”, “monthly to less than weekly” and “weekly or more”. Where we have analysed outcomes over a six year period between assessments, a composite measure of HDO frequency was used in order to capture the extremes of heavy drinking patterns that are most relevant to this research. The three composite HDO categories were: 1. Never or less than monthly, at both assessments; 2. monthly to less than weekly at one or both assessments, but never weekly or more; 3. weekly or more at one or both assessments.

#### Potential confounders

We considered characteristics that could confound the association of drinking pattern and sexual behaviour as plausible common causes of both, based on existing literature. Two important proximal confounders at 26–38 years of age were identified; relationship status and parenting of young children. Given the diversity of blended family situations we restricted the parenting variable to biological children under 6 years of age, to identify situations where most impact was likely to be seen. We also adjusted for socioeconomic status and a history of childhood sexual abuse as these longer-term characteristics have been previously shown to be related to both alcohol and sexual behaviour.

Relationship status: Relationship with a current opposite sex partner was classified as ‘married’, ‘cohabiting’ (living together but not married), ‘regular sexual partner’ (but not living together), or ‘no regular sexual partner’ (current partner(s) not regular, or no current relationship), at age 32 and 38 assessments.

Parenting of a biological child under 6: This was defined as living with such a child, or living apart but sharing care of a child, at age 32 and 38 assessments. The comparison group were those without any biological children under six years old, or where the child(ren) lived full-time with another guardian.

Childhood sexual abuse: “Any childhood sexual abuse” was defined as experiencing unwanted sexual activities before the age of 16, as reported at 26 years of age, with only contact abuse considered. A detailed description of this variable has been published by van Roode *et al*. (2009) [27].

Socioeconomic status (SES): Adult SES was assessed at both 32 and 38 years of age, using the New Zealand Socio-Economic Index [28]. The six categories (‘1’ = unskilled labourer to ‘6’ = professional) were combined (High SES = 5/6, Mid SES = 3/4, Low SES = 1/2) when included in models.

#### Analysis

All statistical analyses were performed using Stata Version 13.1 [29]. For past-year outcomes, we identified and excluded women who were pregnant in the year preceding the assessment, on the basis that this would be likely to affect drinking behaviour in the short-term. Study members who reported no sexual partners during the relevant time period (i.e. past year or past 6 years) were excluded from those analyses.

Distributions of characteristics of the study sample, and of self-reported use of alcohol in relation to sexual activity, were described separately for men and women.

Logistic regression with complete case analysis was used to model associations of HDO frequency with binary outcomes separately for men and women, with unadjusted and adjusted models fitted for each.

Models of the associations between HDO frequency and alcohol use in sexual relationships over the past year at age 38 were adjusted for relationship status, socioeconomic status, and parenting of biological children under six years old at age 38. Models of associations between past-year HDO frequency and past-year sexual partner numbers at ages 32 and 38 were adjusted for relationship status and socioeconomic status measured at age 32 and 38 respectively. Models of associations between HDO frequency for the 6 years prior to the age 32 and age 38 assessments and TOPs or STIs in the same period were adjusted for relationship status and socioeconomic status measured at age 32 and 38 respectively.

We included a history of child sexual abuse (CSA) in a set of corresponding preliminary models and found that while it was independently associated with some outcomes, it did not substantially confound any of the models. We have therefore omitted CSA from our models, since missing data on CSA (4% of men; 3.2% of women) would reduce the number of complete cases and possibly introduce bias.

## Results

Of the survivors of the original cohort of 1037, 94.8% (n = 966) completed the sexual and reproductive health questionnaire at age 26, 94.5% (n = 959) at age 32, and 92.9% (n = 936) at age 38, slightly fewer than for the study as a whole. There were 436 men and 430 women study members who self-identified as heterosexual and reported mostly opposite sex sexual experiences at age 32, and 433 men and 434 women at age 38.

The characteristics of the sample at the age 32 and 38 assessments are shown in Table 1. At age 38, 80.4% of men and 78.6% of women were married or cohabiting with a partner, and 41.8% of men and 46.3% of women were parenting at least one child under 6 years old.supp

**Table 1:** Characteristics of the samples at ages 32 and 38 (%).^a^

	AGE 32		AGE 38	
	Men (*n* = 436)	Women (*n* = 430)	Men (*n* = 433)	Women (*n* = 434)
**Relationship status**				
Married	37.6	49.5	56.4	59.2
Cohabiting	34.6	25.3	24.0	19.4
Regular sexual partner (not cohabiting)	8.9	8.8	5.3	7.6
No regular sexual partner	18.8	16.0	14.3	13.8
Missing	0.0	0.2	0.0	0.0
**Socioeconomic status (NZSEI)**				
High (5/6)	20.0	16.5	26.8	34.6
Mid (3/4)	50.9	55.3	48.5	53.7
Low (1/2)	29.1	28.1	24.5	11.3
Missing	0.0	0.0	0.2	0.5
**Parenting of biological child <6 years old**				
No	—	—	56.6	53.0
Yes	—	—	41.8	46.3
Missing	—	—	1.6	0.7
**No. sexual partners (past yr)**				
0	3.7	3.7	4.4	4.1
1	66.1	80.2	75.1	84.1
2	11.5	7.9	6.2	5.5
3–9	16.1	7.4	11.8	5.1
10+	2.5	0.5	2.1	0.7
Missing	0.2	0.2	0.5	0.5
**No. sexual partners (past 6y)**				
0	0.9	0.2	0.9	0.5
1	29.4	47.9	50.6	64.5
2	11.2	13.5	8.5	11.5
3–9	38.3	30.5	28.2	19.6
10+	18.6	6.7	10.6	2.3
Missing	1.6	1.2	1.2	1.6
**Any same-sex sexual experience (past 6y)**				
No	95.0	93.5	95.8	94.2
Yes	2.8	5.1	2.3	3.9
Missing	2.3	1.4	1.8	1.8
**Sexually transmitted infection (past 6y)** ^**b**^				
No	80.7	81.6	90.5	89.4
Yes	18.3	17.7	9.2	10.6
Missing	0.9	0.7	0.2	0.0
**Termination of pregnancy (past 6y)**				
No	89.4	90.5	95.2	94.7
Yes	9.6	8.8	4.2	5.3
Missing	0.9	0.7	0.7	0.0
**History of childhood sexual abuse**				
No	89.0	67.7	88.7	67.1
Yes	6.9	29.8	7.2	30.0
Missing	4.1	2.6	4.2	3.0

Columns may not sum to 100.0% because of rounding.

^a^ Study members who self-identified as heterosexual and reported mostly opposite-sex sexual experiences. Those never having had opposite-sex sexual intercourse were excluded (4 men and 2 women at age 32; 2 men and 2 women at age 38).

^b^ Self-reported or HSV2/Chlamydia seroconversion.

Men were more likely to report a heavy episodic drinking pattern than women, and to be assessed as alcohol dependent, at both the age 32 and 38 assessments (Table 2). The proportion of cohort members drinking heavily at least weekly, and the proportion diagnosed as alcohol dependent, increased slightly from age 32 to age 38, for both men and women.

**Table 2:** Frequency of heavy drinking occasions (HDOs) and prevalence of alcohol dependency, at 32 and 38 years (%).

	AGE 32		AGE 38	
	Men (*n* = 436)	Women (*n* = 430)	Men (*n* = 433)	Women (*n* = 434)
**HDO frequency (past yr)**				
Never	11.2	33.7	19.6	34.6
Less than monthly	33.9	40.0	33.3	38.5
Monthly to <weekly	33.5	19.3	23.6	16.8
Weekly or more	21.3	7.0	23.6	10.1
**HDO frequency (past 6 yrs)** ^a^				
Never/<monthly	24.1	54.2	34.4	60.8
Monthly to <weekly	35.1	28.1	32.1	24.7
Weekly or more	39.9	17.4	32.6	13.8
Missing	0.9	0.2	0.9	0.7
**Alcohol dependency (past yr)**				
No	89.7	95.1	88.2	94.0
Yes	9.9	4.4	11.5	6.0
Missing	0.5	0.5	0.2	0.0

Columns may not sum to 100.0% because of rounding.

^a^A composite measure based on past-year HDO frequency at ages 26 and 32, or 32 and 38.

### Descriptions of alcohol use in sexual relationships

Self-reported involvement of alcohol in sexual behaviour over the past year is summarised in Table 3. Approximately half of men and women reported that they or their partner drank alcohol before or during sex “rarely” or “never”, with 8.2% of men and 14.6% of women reporting this happened “usually” or “always”. Almost 80% of those who reported any drinking said it was usually both partners drinking. When it was just one partner drinking it was more often the man.

**Table 3:** The role of alcohol in past-year sexual behaviour at age 38, by sex (%).^a^

		Men	Women
	**How often did you or your sexual partner drink alcohol before or during sex?** ^b^	*(n = 413)*	*(n = 377)*
	Never	19.6	15.9
	Rarely	30.3	29.7
	Sometimes	41.9	39.8
	Usually	7.7	13.0
	Always	0.5	1.6
	**Who usually drank alcohol before or during sex?** ^c^	***(n = 332)***	***(n = 313)***
	Self only	17.5	8.3
	Partner only	5.1	11.8
	Both	77.4	79.9
	**On average, when** **you** **drank alcohol before sex, how strongly were you affected?**	***(n = 315)***	***(n = 276)***
	Not at all	46.7	37.0
	Somewhat	47.9	57.2
	Very strongly	0.0	1.8
	Don't know	5.4	4.0
	**On average, when** **your partner** **drank alcohol before sex, how strongly was your partner affected?**	***(n = 274)***	***(n = 287)***
	Not at all	42.3	38.0
	Somewhat	48.9	54.0
	Very strongly	1.8	2.8
	Don't know	6.9	5.2
	**Have you used alcohol to make it easier to have sex?**	***(n = 316)***	***(n = 280)***
	No	89.2	82.1
	Yes	6.3	13.9
	Don't know	4.4	3.9
**Consequences attributed to respondent's drinking**	**Sex without contraception (when pregnancy not wanted)**	***(n = 315)***	***(n = 280)***
	No	89.2	92.1
	Yes	8.3	7.1
	Don't know	2.5	0.7
	**Sex without a condom to protect against an STI**	***(n = 315)***	***(n = 278)***
	No	89.2	91.7
	Yes	7.9	7.6
	Don't know	2.9	0.7
	**Sex that respondent later regretted**	***(n = 316)***	***(n = 280)***
	No	92.7	93.6
	Yes	7.0	5.7
	Don't know	0.3	0.7
**Consequences attributed to partner's drinking**	**Sex without contraception (when pregnancy not wanted)**	***(n = 273)***	***(n = 291)***
	No	88.6	91.1
	Yes	7.3	6.9
	Don't know	4.0	2.1
	**Sex without a condom to protect against an STI**	***(n = 273)***	***(n = 288)***
	No	91.9	91.3
	Yes	4.8	6.3
	Don't know	3.3	2.4

Columns may not sum to 100.0% because of rounding.

^a^Study members with no past-year sexual partners and women with past-year pregnancies were excluded.

^b^Study members who responded 'never' were not asked remaining questions (82 men and 60 women).

^c^Study members who responded 'self only' (58 men and 26 women) or 'partner only' (17 men and 37 women) were not asked questions about the role of alcohol consumption by the respondent or the respondent's partner, repectively.

Women were twice as likely to report that they had used alcohol to make it easier to have sex in the past year compared with men (13.9% vs 6.3%). Further questions put to those using alcohol to make sex easier revealed that about 30% of the women (11/39) and the men (6/20) said they did this “quite a few times” or “often”. More women than men reported that this alcohol use was to make it easier to have sex with a new partner (15/39 vs 5/20), but differences were not statistically significant.

The degree to which respondents were affected by alcohol when they were drinking before sex was reported as “not at all” by 46.7% of men and 37% of women and “somewhat” for most of the remainder (47.9% and 57.2%). A small proportion of women (1.8%) reported that they were very strongly affected, but no men. Reporting of the effect of their partner drinking showed a similar pattern, with 1.8% of men and 2.8% of women reporting that drinking had a very strong effect on their partner.

Similar proportions of men and women reported at least one unwanted consequence of their own or their partner’s drinking before having sex (13.5% of men and 11.9% of women). Approximately 8% of men and 7% of women reported that they had failed to use contraception (when pregnancy was not wanted), or a condom to prevent STIs, due to their drinking in the past 12 months. Of these respondents, approximately 30% of men and 40% of women reported that their alcohol-attributed failure to use contraception (men: n = 8; women: n = 8) or a condom (men: n = 8; women: n = 9) had happened “quite a few times” or “often”.

Seven per cent of men (n = 22) and 6% of women (n = 16) reported having sex they later regretted due to drinking at the time, of whom 2 men and 2 women reported this happening ‘quite a few times’. Almost all of the men (21/22) and women (14/16) said the regret related to choice of partner. Some respondents also reported sex without contraception (7% of men and women), or sex without a condom (5% of men and 6% of women) due to their partner’s drinking at the time.

### Associations of usual drinking patterns with reported alcohol involvement in sex

Table 4 summarises the associations of the self-reported characteristics and consequences of drinking before sex (previously described in Table 3), with respondents’ usual drinking patterns, characterised as frequency of heavy drinking occasions. HDO frequency was significantly associated with the involvement of alcohol in sex “usually or always”, for both men (aOR = 5.46, 95% CI 2.26–13.23) and women (aOR = 12.31, 95% CI 5.16–29.37) when comparing heavy drinking at least weekly with less than monthly, and also with how strongly affected the respondent usually was. These associations were stronger among women than men, with a clear gradient. Usual heavy drinking frequency of weekly or more was also associated with the respondent’s *partner* being strongly or somewhat affected by drinking before sex for men (aOR = 2.05, 95% CI 1.06–3.98) and for women (aOR = 3.05 (95% CI 1.28–7.27).

**Table 4:** Associations of past-year HDO frequency with self-reported alcohol involvement in sex in the past year.^a^

		Men							Women						
		n	Unadjusted			Adjusted^b^			n	Unadjusted			Adjusted^b^		
			OR	(95% CI)	p	aOR	(95% CI)	p		OR	(95% CI)	p	aOR	(95% CI)	p
	**Usually/always drank alcohol before sex (either partner)**														
	Never/<Monthly HDOs		1.00			1.00				1.00			1.00		
	Monthly to <Weekly HDOs		2.23	(0.81,6.13)	0.120	2.12	(0.76,5.91)	0.150		**4.28**	**(2.08,8.80)**	**<0.001**	**3.50**	**(1.63,7.50)**	**0.001**
	Weekly+ HDOs		**5.86**	**(2.45,14.02)**	**<0.001**	**5.46**	**(2.26,13.23)**	**<0.001**		**10.17**	**(4.75,21.78)**	**<0.001**	**12.31**	**(5.16,29.37)**	**<0.001**
		406							374						
	**Very strongly/somewhat affected when drinking before sex**														
	Never/<Monthly HDOs		1.00			1.00				1.00			1.00		
	Monthly to <Weekly HDOs		1.51	(0.86,2.65)	0.147	1.47	(0.82,2.63)	0.190		**1.95**	**(1.05,3.64)**	0.035	1.92	(1.01,3.66)	0.048
	Weekly+ HDOs		**2.59**	**(1.47,4.57)**	**0.001**	**2.50**	**(1.40,4.47)**	**0.002**		**5.16**	**(2.06,12.93)**	<0.001	5.13	**(1.95,13.46)**	**0.001**
		292							264						
	**Partner was very strongly/somewhat affected when drinking before sex**														
	Never/<Monthly HDOs		1.00			1.00				1.00			1.00		
	Monthly to <Weekly HDOs		1.27	(0.70,2.29)	0.432	1.19	(0.64,2.20)	0.584		1.33	(0.72,2.44)	0.359	1.40	(0.74,2.62)	**0.300**
	Weekly+ HDOs		**2.00**	**(1.06,3.77)**	**0.033**	**2.05**	**(1.06,3.98)**	**0.033**		**3.15**	**(1.37,7.24)**	**0.007**	**3.05**	**(1.28,7.27)**	0.012
		249							271						
	**Used alcohol to make it easier to have sex**														
	Never/<Monthly HDOs		1.00			1.00				1.00			1.00		
	Monthly to <Weekly HDOs		2.17	(0.69,6.87)	0.187	1.97	(0.60,6.47)	0.265		**3.49**	**(1.57,7.75)**	**0.002**	**3.31**	**(1.43,7.69)**	**0.005**
	Weekly+ HDOs		2.05	(0.63,6.68)	0.236	1.97	(0.58,6.72)	0.278		**3.77**	**(1.53,9.30)**	**0.004**	**2.83**	**(1.04,7.67)**	**0.041**
		296							268						
**Consequences attributed to respondent's drinking**	**Sex without contraception (when pregnancy not wanted)**														
	Never/<Monthly HDOs		1.00			1.00				1.00			1.00		
	Monthly to <Weekly HDOs		1.95	(0.60,6.35)	0.268	1.69	(0.49,5.87)	0.408		2.21	(0.79,6.21)	0.132	1.52	(0.50,4.64)	0.460
	Weekly+ HDOs		**4.06**	**(1.39,11.84)**	0.010	**3.20**	**(1.01,10.15)**	**0.048**		1.89	(0.55,6.48)	0.308	1.01	(0.26,3.94)	0.987
		301							277						
	**Sex without a condom to protect against an STI**														
	Never/<Monthly HDOs		1.00			1.00				1.00			1.00		
	Monthly to <Weekly HDOs		1.17	(0.41,3.34)	0.774	0.92	(0.30,2.85)	0.885		2.95	(0.91,9.51)	0.071	1.10	(0.28,4.43)	0.888
	Weekly+ HDOs		1.58	(0.58,4.26)	0.371	1.23	(0.41,3.71)	0.708		**7.50**	**(2.50,22.50)**	**<0.001**	3.26	(0.88,12.12)	0.078
		300							275						
	**Sex that respondent later regretted**														
	Never/<Monthly HDOs		1.00			1.00				1.00			1.00		
	Monthly to <Weekly HDOs		3.35	(0.84,13.32)	0.086	3.37	(0.80,14.25)	0.098		**5.83**	**(1.41,24.05)**	**0.015**	3.12	(0.68,14.30)	0.143
	Weekly+ HDOs		**6.10**	**(1.67,22.30)**	**0.006**	**5.39**	**(1.37,21.25)**	**0.016**		**11.60**	**(2.85,47.12)**	**0.001**	**5.89**	**(1.23,28.30)**	**0.027**
		309							277						
**Consequences attributed to partner's drinking**	**Sex without contraception (when pregnancy not wanted)**														
	Never/<Monthly HDOs		1.00			1.00				1.00			1.00		
	Monthly to <Weekly HDOs		1.42	(0.48,4.24)	0.524	1.21	(0.39,3.78)	0.737		1.78	(0.57,5.53)	0.320	1.36	(0.42,4.42)	0.613
	Weekly+ HDOs		1.24	(0.38,4.07)	0.725	0.97	(0.27,3.43)	0.963		**3.56**	**(1.19,10.66)**	**0.024**	2.53	(0.76,8.42)	0.131
		256							284						
	**Sex without a condom to protect against an STI**														
	Never/<Monthly HDOs		1.00			1.00				1.00			1.00		
	Monthly to <Weekly HDOs		0.86	(0.20,3.73)	0.845	0.77	(0.17,3.51)	0.736		1.92	(0.54,6.81)	0.313	1.22	(0.31,4.80)	0.778
	Weekly+ HDOs		1.37	(0.36,5.30)	0.647	1.26	(0.29,5.44)	0.760		**5.74**	**(1.88,17.51)**	**0.002**	**3.65**	**(1.04,12.82)**	**0.043**
		258							280						

Totals differ due to survey skip patterns, and study members who responded 'don't know' to a given question were excluded from that analysis.

^a^Study members with no past-year sexual partners and women with past-year pregnancies were excluded.

^b^Adjusted for relationship status, socioeconomic status, and parenting of biological children under 6 years old.

Among women there was a strong association of heavy drinking at least monthly with using alcohol to make it easier to have sex. A weaker association was suggested for men but was not statistically significant.

### Associations of usual drinking patterns with consequences attributed to drinking

Among men, weekly+ HDO frequency compared with <monthly HDO frequency was associated with having had sex without contraception when pregnancy was unwanted (aOR = 3.20, 95% CI 1.01–10.2), but this association was not seen in women. Failure to use a condom due to drinking was not associated with HDO frequency in men, but among women there was a strong unadjusted association that was attenuated after adjustment for confounders and no longer statistically significant. In women, weekly+ HDO frequency was associated with reporting sex without a condom due to a partner’s drinking (aOR = 3.65, 95% CI 1.04–12.82).

There was a graded association of HDO frequency with sex that was later regretted due to drinking, for both men and women. The most frequent heavy drinkers (weekly or more) were five times more likely to report regret than respondents whose heavy drinking was less than monthly. The unadjusted association was substantially stronger in women than men (OR = 11.60 vs 6.10) but was more confounded by socioeconomic and relationship status.

### Associations of usual drinking pattern with number of sexual partners, TOPs and STIs

Table 5 shows the associations of HDO frequency with number of sexual partners in the past year, and with TOPs and STIs in the past 6 years at age 38 assessment, and compares them with equivalent analyses at age 32.

**Table 5:** Associations of HDO frequency with number of sexual partners, STIs and TOPs at ages 32 and 38^a^.

	Men							Women						
	n	Unadjusted			Adjusted			n	Unadjusted			Adjusted		
		OR	(95% CI)	p	aOR	(95% CI)	p		OR	(95% CI)	p	aOR	(95% CI)	p
**3+ sexual partners in past year (age 38)** ^b^ ^c^														
** HDO Frequency (past yr)**														
Never/<Monthly		1.00			1.00				1.00			1.00		
Monthly to <Weekly		1.38	(0.71,2.72)	0.344	1.38	(0.66,2.93)	0.394		**2.91**	**(1.07,7.97)**	**0.037**	1.69	(0.55,5.23)	0.363
Weekly+		1.66	(0.86,3.19)	0.132	1.44	(0.70,2.96)	0.323		**4.94**	**(1.77,13.79)**	**0.002**	**4.05**	**(1.16,14.16)**	**0.028**
	412							375						
**3+ sexual partners in past year (age 32)** ^b^ ^d^														
**HDO Frequency (past yr)**														
Never/<Monthly		1.00			1.00				1.00			1.00		
Monthly to <Weekly		**2.76**	**(1.47,5.17)**	**0.002**	**2.71**	**(1.37,5.34)**	**0.004**		**4.72**	**(2.00,11.15)**	**<0.001**	**4.15**	**(1.64,10.50)**	**0.003**
Weekly+		**5.40**	**(2.82,10.34)**	**<0.001**	**4.26**	**(2.09,8.67)**	**<0.001**		**9.40**	**(3.42,25.84)**	**<0.001**	**9.43**	**(3.00,29.64)**	**<0.001**
	419							324						
**Sexually transmitted infection (age 32–38)** ^c^														
**HDO Frequency (32–38 composite)**														
Never/<Monthly		1.00			1.00				1.00			1.00		
Monthly to <Weekly		0.97	(0.38,2.47)	0.950	1.12	(0.43,2.90)	0.820		1.13	(0.53,2.39)	0.756	0.87	(0.39,1.92)	0.731
Weekly+		**2.40**	**(1.09,5.29)**	**0.030**	**2.37**	**(1.04,5.39)**	**0.040**		**2.21**	**(1.01,4.80)**	**0.046**	1.57	(0.69,3.59)	0.281
	424							427						
**Sexually transmitted infection (age 26–32)** ^d^														
**HDO Frequency (26–32 composite)**														
Never/<Monthly		1.00			1.00				1.00			1.00		
Monthly to <Weekly		1.08	(0.58,2.03)	0.810	1.13	(0.59,2.15)	0.712		1.44	(0.82,2.55)	0.206	1.35	(0.75,2.44)	0.318
Weekly+		0.87	(0.46,1.63)	0.659	0.76	(0.40,1.45)	0.405		1.41	(0.72,2.76)	0.317	1.36	(0.68,2.75)	0.388
	424							424						
**Termination of pregnancy (age 32–38)** ^c^														
**HDO Frequency (32–38 composite)**														
Never/<Monthly		1.00			1.00				1.00			1.00		
Monthly to <Weekly		1.91	(0.55,6.68)	0.310	2.03	(0.57,7.21)	0.273		0.43	(0.09,1.98)	0.279	0.36	(0.08,1.72)	0.202
Weekly+		1.88	(0.54,6.58)	0.322	1.67	(0.47,5.95)	0.429		**4.53**	**(1.82,11.23)**	**0.001**	**3.81**	**(1.47,9.90)**	**0.006**
	422							427						
**Termination of pregnancy (age 26–32)** ^d^														
**HDO Frequency (26–32 composite)**														
Never/<Monthly		1.00			1.00				1.00			1.00		
Monthly to <Weekly		1.41	(0.55,3.63)	0.474	1.56	(0.59,4.11)	0.365		1.56	(0.75,3.26)	0.235	1.33	(0.62,2.85)	0.465
Weekly+		1.92	(0.79,4.69)	0.152	1.61	(0.64,4.01)	0.311		1.06	(0.40,2.78)	0.906	0.85	(0.32,2.31)	0.754
	424							424						

^a^ Each analysis excludes study members reporting no sexual partners in the relevant period.

^b^ Women with past-year pregnancies were excluded.

^c^ aORs adjusted for relationship status and socioeconomic status at age 38.

^d^ aORs adjusted for relationship status and socioeconomic status at age 32.

Among 38 year old men there was no association observed between frequency of heavy drinking and having three or more sexual partners in the last year. However in women there was a graded association with partner numbers that was attenuated by adjustment for relationship status and socioeconomic status, but remained strong for the weekly+ heavy drinkers (OR for 3+ sexual partners 4.05; 95% CI 1.16–14.16). At age 32, the association of heavy drinking frequency with number of sexual partners in the past 12 months was strong and graded for both men and women, and largely independent of relationship status and socioeconomic status.

Despite the strong associations of heavy drinking with partner numbers, the only significant association of drinking pattern with STIs was seen in men during the 32–38 year period.

Women who were weekly+ heavy drinkers at either age 32 or age 38 were four times as likely to have had a termination of pregnancy in the period 32–38 years compared with women who reported heavy drinking less than monthly at both assessments. There was no significant association between drinking pattern of male respondents from 32 to 38 and pregnancy termination by a partner in those 6 years, and no association between drinking pattern and termination of pregnancy in the period 26–32 years for men or women.

## Discussion

These findings show that sex was commonly accompanied by drinking in this sample of 38 year olds in a New Zealand birth cohort. Heavy drinking patterns were associated with more sexual partners, regretted sex, STIs and pregnancy terminations. Gender differences suggest that drinking in this context may be more important for women.

About half of men and women aged 38 reported drinking before or during sex more than “rarely” in the last year, with 8.2% of men and 14.6% of women reporting “usually” or “always”. Similar proportions of men and women (6–8%) attributed failure to use contraception, condom non-use, and regretted sex to their own drinking in the preceding 12 months. Failure to use condoms due to their own drinking, and women’s reports of lack of contraception due to their own drinking were not significantly associated with usual drinking pattern after adjustment for confounders, but failure to use contraception was more commonly reported by heavy drinking men. Usual frequency of heavy drinking was associated with usually or always drinking before or during sex and with regretted sex, after adjustment for relationship status, SES, and parenting of a biological child under 6. Regret about sex attributed to drinking was almost always due to poor partner choice. Women reporting a heavy drinking occasion at least once a month were three times more likely to report using alcohol to make it easier to have sex.

Associations of HDO frequency with numbers of sexual partners, STIs and TOPs showed gender differences and also changes over time. Past-year partner numbers increased with heavy drinking frequency in men and women at age 32, the association being stronger in women. At 38 years, no association with partner numbers was seen for men, but it persisted in women even after adjustment for relationship status and SES. Heavy drinking at least weekly was also associated with pregnancy termination during past 6 years for women aged 38, but not in the earlier period.

A strength of this study is that it is a population-based birth cohort, and so it is possible to examine differences by age in the same people. It also means we have identical alcohol consumption measures at relevant time points, avoiding retrospective accounts of drinking pattern which are demonstrably unreliable [30], and we have data on important confounders. In addition, the cohort has a very high retention rate, reducing the potential non-response biases which may affect many studies of young adult drinking [31]. The high retention and good representativeness of the New Zealand population allows cautious generalisation to similar countries. Using the alcohol measure most appropriate to the expected mechanism of a causal effect of alcohol on sexual behaviour, rather than depending on measures of average consumption or alcohol use disorders [7], also increases confidence in the findings.

One important limitation is that most of the data are self-reported and apart from potential for non-disclosure, self-reporting of STI diagnoses in particular can be inaccurate [25].

The findings presented are also largely descriptive. While there is a clear underlying hypothesis that the associations seen are at least partly causal through intoxication, some potential confounders are not included in these analyses. The birth cohort provides only a modest sized sample, but the homogeneity of the sample in terms of age means that it can more strongly represent this age group than many large surveys [32].

There are interesting gender differences in these findings. Taken together it appears that women’s drinking is more closely related to the sexual behaviours and outcomes we have measured than men’s drinking. The association between usual drinking pattern and drinking with sex was stronger in women, and women reported being more strongly affected. While the prevalence of both heavy drinking and multiple sexual partners was higher among men, the association between drinking and partner numbers was consistently stronger for women. The Natsal studies reported similar findings for partner numbers from representative surveys of 16–44 year olds in the UK using a different heavy drinking measure. The association was twice as strong in women as men in both 1990 and 2000 [12]. It has been suggested previously that explanations for gender differences may be that women experience more dissonance between these behaviours and their own and societal expectations than men do, that motivations for sex vary by gender, and that men are generally more accepting of casual sex [3]. Supporting this argument, data from our cohort show large gender differences in opinions about sex outside of relationships. At age 38, 37% of men and 20% of women reported that “one-night stands” were “rarely wrong/not wrong at all”, and there were similar gender differences in opinions about non-exclusivity in relationships generally [33]. The current study shows women were twice as likely as men to report using alcohol to make it easier to have sex, and this was related to usual drinking pattern in women but not men. This is consistent with previous studies describing women’s use of alcohol for its disinhibiting effects in sexual situations that create anxiety [34 35]. Frequent heavy drinking women were also more likely than other women to have terminated a pregnancy in the six years from age 32 to 38, once relationship status, SES and number of partners had been taken into account, but this was not so for heavy drinking men.

Some care needs to be taken in interpreting the magnitude of gender differences as both consumption and outcomes were self-reported. Gender differences in recall of behaviour or outcomes may arise from differences in salience for men and women, or even differences in knowledge with regard to terminations of pregnancy. Men and women may also differ in their willingness to disclose, given that the sensitivity of the questions may vary by gender and by drinking status [36].

We saw a stronger association between usual drinking and partner numbers at 32 than 38 for both men and women, but no association of usual drinking with STIs or pregnancy terminations over the six years from 26 to 32, which contrasted with the later period. The Natsal-2 study also found no association of STI diagnosis with their measure of heavier drinking (drinking above the guidelines) and an “equivocal” relationship of heavy drinking with abortion, but it included adults of all ages and so is not directly comparable [12]. Our findings with regard to STIs differ from those of another NZ cohort analysis, where level of alcohol consumption was found to be causally associated with STI diagnosis. However, there were substantial differences in alcohol measure and age groups included, and no gender-specific analysis [7].

The prevalence of regular heavy drinking in the two age periods was similar. However, the patterns of association with outcomes differed by age, demonstrating the importance of context when discussing links between alcohol and sexual behaviours [2]. Reasons for the differences are unclear, but the comparison combines age and period effects. Both age and period are likely to affect the dominant norms of behaviour, and therefore the nature of important confounders of these associations, even in the same population.

The lack of association between heavy drinking frequency and failure to use a condom is consistent with studies conducted amongst university students, where usual drinking patterns are associated with drinking before sex, but condom use when intoxicated is strongly associated with condom use when sober [8 37]. The 1994 U.S. general population survey also found no association between drinking pattern and condom use in respondents with more than one partner in the previous 12 months [20]. The lack of effect on condom use helps explain why STIs are not as strongly related to heavy drinking as are partner numbers.

In conclusion, the findings indicate that alcohol involvement in sexual behaviour and its consequences continues beyond the period of young adulthood where it has been well documented, and that women appear to be more affected than men. The British Natsal studies have documented substantial change in sexual lifestyles since 1990 [32] and there has been coincident change in drinking patterns in the United Kingdom, New Zealand and similar countries, particularly affecting women. A commitment to improving sexual health and wellbeing throughout the life course needs to take account of the potential role of alcohol in sexual behaviour and its consequences.
